# Report of a Case of Gun-Shot Wound, with Observations

**Published:** 1856-09

**Authors:** Walter Somerville

**Affiliations:** Culpepper County, Virginia


### ARTICLE IV.

*Report of a Case of Gun-shot Wound, with observations.* By WALTER SOMERVILLE, M. D., Culpepper County, Virginia.

"Many a limb is sacrificed by the catling and saw that might be saved by the use of a more sober and cautious judgment."—*Meigs.*

The vis medicatrix naturæ appears wonderful to us, in many of those diseases which come within the province of the physican, but still more so in many of those injuries which belong to the department of the surgeon.

The eloquent writer quoted above, remarks, in connection with this subject, "doubtless, in the history of surgery, many cases are to be found of operations performed without necessity." Of the truth of this remark, no one possessing any experience in, or any acquaintance with the treatment of surgical cases, can doubt; yet we do meet with cases in which it becomes a very important and responsible duty to determine whether or not amputation ought to be resorted to. Of such a character was the case which I now propose to report, and the result of which is calculated to encourage us in the pursuit of that course in which I think we need encouragement, namely, to estimate more highly, and trust more than we have done, to this important principle, the vis medicatrix naturæ, or as it would be more appropriately expressed, the vis medicatrix dei.

On the 31st of May, of the present year, I was called to visit a colored boy, about 13 years of age, belonging to Mr. W. W., of this county, who, in attempting to raise a gun from the corner of a fence, on the side opposite to that on which he was, the cock, coming in contact with a rail, was drawn back, and falling upon the tube, the gun discharged, and the muzzle being held by the right hand, the load consisting of large squirrel shot, entered the hand near the centre of its palm, and passing obliquely upwards and outwards, made its exit through the upper and back part of the hand and the lower part of the wrist joint. By recollecting the manner in which we naturally take hold of the head of a walking cane for the purpose of using it, applying the palm of the hand to the head of the cane, we shall readily comprehend the course of the wound. However, to be more accurate, it entered rather above the centre of the palm, and passed out between the metacarpal bones of the index and ring fingers and that part of the wrist joint contiguous, fracturing the upper part of the metacarpal bone of the middle finger and several of the bones of the wrist.*

By probing the wound with my ring finger, I soon discovered, as I had supposed, that the whole of the load, shot and wadding, had passed out.

The point of difficulty and responsibility in this case, was in deciding whether or not amputation should be resorted to. The advice of most authors, is to amputate in gun-shot wounds seriously affecting the joint, yet bearing in mind the progress of surgical science, and influenced somewhat by my own experience, I decided against this resort. This was a case, however, in which the life of a fellow creature being concerned, I considered consultation desirable, and obtained the advice of my very judicious and experienced friend, Dr. Holladay, of Orange county. He concurred with me, and the result is, that after the lapse of ——— since the injury was received, the patient is now entirely free from danger; the wound has almost entirely healed, and the patient will regain almost perfect use of the hand and fingers. The treatment adopted was simple.

* Six pieces of bone, mostly about the size of that part of the nail which appears on the little finger, have been discharged by the sloughing and suppurating process.

Cold water dressings frequently renewed during the first 36 or 48 hours; fluid opium to allay nervous irritation, and procure sleep; low diet, with purgatives during the period of fever; and during the sloughing stages, poultices, especially the fermenting poultice, with olive oil or lard incorporated, and freely applied to the surface, gently washing morning and evening with mild soap and water, and a dilute solution of chloride of soda after each ablution, embraced the whole of the treatment, to which should have been added an elevated position and rest of the part.

